# Correlations Between Rheology, In Situ Mucosal Retention and In Vivo Immunogenicity Reveal the Potential and Limitations of Mucoadhesive Excipients for Sublingual Vaccine Delivery

**DOI:** 10.3390/pharmaceutics17111456

**Published:** 2025-11-11

**Authors:** Mohamed Deifallah Yousif, Ilona Kubajewska, Fatme Mawas, Sudaxshina Murdan

**Affiliations:** 1 UCL School of Pharmacy, University College London, 29-39 Brunswick Square, London WC1N 1AX, UK; mohamed.yousif@ucl.ac.uk (M.D.Y.); i.kubajewska@ucl.ac.uk (I.K.); 2 Vaccines Division, Scientific Research & Innovation Group, MHRA, Potters Bar EN6 3QG, UK

**Keywords:** sublingual delivery, vaccine, mucosal immunity, immunogenicity, hydroxypropyl methylcellulose (HPMC), chitosan (MGC), mucoadhesive, polymers, excipients, correlations

## Abstract

**Background/Objectives**: Sublingual vaccination offers a non-invasive route for inducing both systemic and mucosal immunity, yet the formulation properties that govern its success remain poorly defined. This study investigated the relationships among key formulation parameters for sublingual vaccines, such as viscosity, mucoadhesion, and mucosal residence, to understand their impact on in vivo immune responses in the sublingual delivery context. **Methods**: Ovalbumin (OVA)-based vaccine formulations containing cholera toxin B (CTB) adjuvant and mucoadhesive excipients such as hydroxypropyl methylcellulose (HPMC) or methylglycol chitosan (MGC), were evaluated for: (1) their respective rheological properties—characterized by viscosity and mucoadhesion parameters, as well as (2) in situ mucosal retention (assessed using Cy7-labeled formulations tracked by IVIS in vivo imaging system) and (3) in vivo immunogenicity via systemic (IgG) and mucosal (IgA) responses measured by ELISA, following sublingual administration to mice. Correlations between rheology, in situ/ex situ mucosal residence, and in vivo immune outcomes were determined. **Results**: Sublingual vaccine formulations containing HPMC exhibited the highest viscosity, mucoadhesion, and mucosal retention profiles, but paradoxically elicited the weakest systemic and mucosal antibody responses. In contrast, chitosan-based formulations enhanced immune responses even at reduced antigen and adjuvant doses, likely due to its permeation-enhancing and adjuvant effects. Correlation analyses revealed that while formulation viscosity and mucoadhesive strength were positively associated with mucosal retention, both rheological and retentive properties showed a significant inverse relationship with immunogenicity in the context of sublingual vaccine delivery. **Conclusions**: While viscosity and mucoadhesion are essential for in situ retention of sublingual vaccines, prolonged residence driven by excipient’s excessive rheological strength was found to reduce vaccine immunogenicity—likely due to restricted antigen release and mucosal uptake. Accordingly, HPMC appears suboptimal as a sublingual vaccine excipient, while chitosan shows promise for sublingual delivery as a permeation-enhancing adjuvant. These findings may shift the design paradigm for sublingual vaccine formulations, highlighting the need to balance mucosal retention with efficient antigen absorption for maximizing immune responses.

## 1. Introduction

Sublingual administration has emerged as a promising route for vaccine delivery, capable of inducing both systemic and mucosal immune responses while circumventing invasive procedures and first-pass metabolism. This approach has demonstrated protective humoral and cell-mediated responses across a range of mucosal sites—including the oral cavity, nasal passages, throat, trachea, lungs, urogenital system, and to a lesser extent in the gastrointestinal tract [1,2,3,4,5,6].

This capacity for inducing protection at multiple mucosal sites highlights the potential of sublingual immunization against diverse pathogens, including influenza virus, severe acute respiratory syndrome (SARS), human immunodeficiency virus type 1 (HIV-1), human papillomavirus (HPV), Streptococcus pneumoniae, and Group A Streptococcus [1,7,8,9,10,11,12,13].

Beyond its immunological breadth, the sublingual route offers practical advantages: non-invasiveness, needle-free administration, possibility of self-dosing, rapid onset of action—all of which contribute to reduced healthcare costs, enhanced vaccine accessibility and patient compliance [14,15]. However, its widespread clinical adoption is hindered by several limitations, including the tolerogenic nature of the oral mucosal environment [16,17], and rapid clearance of liquid formulations by salivary flow and the swallowing reflex. These factors reduce antigen–mucosa contact time, limit immune cell engagement, and contribute to variability in vaccine efficacy in terms of immune outcomes.

To address these barriers, recent research has focused on optimizing both antigen delivery systems and formulation matrices. Strategies include: (i) the use of potent mucosal adjuvants—such as cholera toxin B-subunit (CTB), monophosphoryl lipid A (MPLA), resiquimod or methylglycol chitosan (MGC), to enhance immune activation; (ii) advanced antigen carriers including liposomes, polymers, adenoviral vectors, virosomes, virus-like particles, or microneedles; and (iii) surface modification of antigen constructs, e.g., via PEGylation or PASylation (using polyethylene glycol or short sequences of Proline, Alanine and Serine amino acids, respectively) to overcome the mucus barrier and facilitate epithelial penetration [15,18,19,20,21,22,23,24,25,26]. Additionally, needle-free injectors [27], semi-solid and solid dosage forms—including mucoadhesive films, gels [26,28,29,30,31] and tablets [32,33] –have been explored as alternatives to early liquid-based sublingual immunization formulations to prolong antigen residence and improve mucosal contact [15,34,35]. Despite these advances, only one sublingual vaccine—Uromune^®^ for recurrent urinary tract infections—has been commercialized to date [36]. This highlights the underexplored nature and nascent stage of sublingual vaccine development relative to more established mucosal routes such as oral or intranasal immunization. The fundamental determinants of effective sublingual immunization have yet to be fully characterized.

The present study addresses this critical gap by examining the interplay between vaccine formulation rheology and its mucosal residence, in relation to in vivo immune outcomes in a murine model following sublingual delivery. We investigated these properties using viscosity and mucoadhesion excipients like hydroxypropyl methylcellulose (HPMC) [37,38] and methylglycol chitosan (MGC) [21,25] in sublingual vaccine formulations, containing ovalbumin (OVA) as a model antigen, and CTB as a mucosal adjuvant [39,40,41,42]. By correlating formulation viscosity, mucoadhesion, and in situ retention with systemic and mucosal in vivo antibody responses it generated, we sought to identify key formulation parameters influencing sublingual vaccine efficacy. Our findings provide insights into formulation-dependent limitations within the sublingual context—for example, that high rheological and retentive capacity does not translate to enhanced immunogenic potency—overall revealing fundamental design considerations for the optimization of future sublingual vaccine platforms.

## 2. Materials and Methods

### 2.1. Materials

Ovalbumin (OVA) chicken egg grade VI, methylglycol chitosan (MGC), hydroxypropyl methylcellulose (HPMC 2910; H7509/25G MW 86 kDa), propylene glycol (PG), recombinant cholera toxin B-subunit (CTB), Tween 20, bovine serum albumin (BSA), phosphate-buffered saline (PBS) were purchased from Sigma-Aldrich (Dorset, UK). Protease inhibitor buffer (PIB) tablets, 3,3′,5,5′ tetramethylbenzidine (TMB ELISA substrate), ethylenediaminetetraacetic acid (EDTA), and foetal bovine serum (FBS) were obtained from Fisher Scientific (Loughborough, UK). Cy7 NHS ester (non-sulfonated) fluorescent dye was sourced from Generon (Slough, UK). Goat anti-mouse IgG-HRP was purchased from Abcam (Cambridge, UK) and goat anti-mouse IgA-HRP from Invitrogen (Carlsbad, CA, USA). Fresh porcine buccal mucosa was obtained from a local abattoir. PBS-T wash buffer (0.5% *v/v* Tween 20 in PBS), assay diluent (PBS + 1% BSA + 0.01 M EDTA + 0.5% Tween 20), and protease inhibitor buffer–PIB (49 mL deionized water + 1 mL FBS + 1 PIB tablet) were prepared in-house.

### 2.2. Animals and Ethics

All animal procedures were conducted in accordance with the United Kingdom Home Office Scientific Procedures Act (1986). A total of sixty female BALB/c mice (8–12 weeks old, ~20 g) were obtained from Charles River Laboratories, UK, and housed under pathogen-free conditions with free access to food and water, and a 12 h light/dark cycle. Animals were acclimatized for one week prior to experimentation. Euthanasia was performed using carbon dioxide inhalation, followed by cervical dislocation to ensure death.

### 2.3. Preparation of Vaccine Formulations

Stock solutions of OVA and chitosan were prepared by dissolving in deionized water. HPMC gel was formulated by adding 50 mg MGC, 600 mg HPMC, 5 mL PG, and 3 mL ethanol (70% *v*/*v*) to 2 mL deionized water under continuous stirring (900–1000 rpm) for 15 min. The mixture was then incubated in a water bath for 30 min at 25 °C.

Seven vaccine formulations (A–G) were prepared by combining OVA, CTB, MGC, and HPMC according to amounts given in Table 1. All formulations were tested for immunogenicity, while selected formulations were further analyzed for sublingual residence, viscosity, and mucoadhesion (Table 1).

### 2.4. In Vivo Immunization Study

#### 2.4.1. Immunization Protocol

Thirty-five mice were randomly assigned to seven groups (n = 5 per group) corresponding to vaccine formulations A–G. Mice were anesthetized with isoflurane prior to immunization, and subsequently administered 10 μL of the respective formulation sublingually on days 1, 8, 15, 22, and 29, following a previously optimized and published immunization protocol [35,43]. Viscous formulations (C, F, G) were delivered using a positive displacement pipette with capillary piston tips (Gilson MICROMAN™, Middleton, WI, USA).

#### 2.4.2. Sample Collection

Blood samples were collected on days 0 and 21 via lateral tail vein, or by cardiac puncture at the study endpoint on day 36. Serum was separated by blood centrifugation at 13,000 rpm for 10 min. Tissues and mucosal washes were harvested post-sacrifice on day 36. Oral washes were obtained by flushing the oral cavity with 50 μL PIB (3 repetitions per mouse). Similarly, vaginal lavage fluid was collected by flushing the vaginal cavity. Intestinal specimens were prepared by excising and fragmenting small intestines into small sections in 2 mL PIB, followed by centrifugation and supernatant collection.

#### 2.4.3. Anti-OVA Antibody Quantification by ELISA

OVA-specific serum IgG and mucosal IgA in oral or vaginal washes and intestinal samples were measured by ELISA, adapting a previously published in-house protocol [35,43]. High-binding 96-well plates (Nunc^TM^ MaxiSorp^TM^, Nunc, Roskilde, Denmark) were coated with 100 μL/well of OVA (10 µg/mL in bicarbonate buffer, pH 8.2) and incubated at 4 °C overnight. After washing three times with PBS-T, blocking with PBS supplemented with 1% *w*/*v* BSA, 0.01 M EDTA, and 0.5% *v*/*v* Tween 20 at 37 °C for 30 min., and washing again, serial dilutions of serum or mucosal samples were added. For IgG, 200 μL of murine sera (1:100 dilution) was added to the first column, followed by serial two-fold dilutions in the remaining columns across the plate. For IgA, 200 μL of undiluted intestinal samples or 100 μL of two-fold diluted oral/vaginal washes were added, followed by serial dilutions as above. Plates were incubated at room temperature (RT) for 2 h, washed, then incubated with goat anti-mouse IgG-HRP (100 μL/well of 1:2000 dilution) or IgA-HRP (100 μL/well of 1:1000 dilution) detection antibodies at RT for 1.5 h. After washing, 100 μL of pre-warmed TMB substrate was added and incubated at RT for 10–15 min. Reactions were stopped with 50 μL of 3 M HCl per well, and absorbance was read at 450 nm using a SpectraMax M2e microplate reader (Molecular Devices, Wokingham, UK). Antibody titres were expressed as endpoint dilution titres—defined as the highest dilution yielding an OD value of 0.5 above background—which is consistent with standard immunogenicity reporting in murine vaccine studies. All samples were assayed in duplicate, and data are presented as antibody titres for individual mice and geometric mean titres per group (n = 5).

### 2.5. In Vivo Imaging of Mucosal In Situ Residence and Its Spatiotemporal Clearance Kinetics Using IVIS

The in vivo residence of vaccine formulations upon sublingual administration in mice was imaged and quantified using IVIS^®^-Spectrum system (Xenogen-Caliper Life Sciences, Alameda, CA, USA). Twenty-five mice were randomly assigned to five test groups (n = 5 mice per group). Following anesthesia (induction with 3–4% isoflurane in the anesthetic chamber), each mouse was sublingually administered with 10 μL of respective formulation containing 200 μg Cy7 fluorescent dye replacing OVA to enable visualization and tracking of the formulation. Imaging was performed under anesthesia (~2% isoflurane) at 0, 2, 4, 8, 20, and 28 h post-administration, and fluorescence intensity in the excitation/emission wavelengths corresponding to Cy7 signal (740/790 nm) was recorded. IVIS imaging settings were as follows: lamp level = high, binning = medium (4), field of view (FOV) = 24, f = 1.2, exposure time = 5 s. The images were analyzed using the IVIS Living Image^®^ 3.0 software (PerkinElmer, Waltham, MA, USA). The min. and max. signal levels were adjusted to the same scale for each image to be normalized across all data. Circular regions of interest (ROI) were defined over the oral cavity or gastrointestinal tract areas and replicated across images for direct comparison. Cy7 fluorescence intensity in the respective ROIs was then quantified by the software as epifluorescence counts. 

Total retention of Cy7 signal over time was quantified using trapezoidal integration of fluorescence intensity values obtained from IVIS imaging. For each animal, the area under the curve (AUC) was calculated across sequential time intervals using the trapezoid rule:

AUC_i_ = [(I_i_ + I_i+1_)/2] × (t_i+1_ − t_i_)

where I_i_ and I_i+1_ are fluorescence intensities corresponding to timepoints t_i_ and t_i+1_.

The total AUC was then computed as the sum of all interval-specific AUCs:

Total AUC = Σ AUC_i_

This method assumes linear interpolation between measurements and provides a cumulative estimate of retention across the full imaging period. Time intervals were defined as follows: 0–2 h, 2–4 h, 4–8 h, 8–20 h, and 20–28 h. AUC values were computed individually for each mouse and used to compare retention kinetics across formulations. This approach has been reported for analysis of pharmacokinetic and biodistribution data, including fluorescence-based imaging studies [44].

### 2.6. Formulation Rheological Characterization

Vaccine formulations C, E, and G (corresponding to formulations listed in Table 1) were subjected to the following rheological characterization.

#### 2.6.1. Viscosity Measurements

Viscosity was measured using a Bohlin Gemini HR Nano Rheometer (Malvern Panalytical Ltd., Malvern, UK) equipped with a cone-and-plate geometry (5998/J01/31 4/40 plate). Measurements were conducted at 25 °C in isothermal mode with a fixed gap height of 150 μm. Shear rates ranged from 4000 to 45,000 s^−1^, with a delay and integration time of 5 s per measurement. Each formulation was tested in triplicate, and six viscosity readings at different shear rates were recorded per sample.

#### 2.6.2. Mucoadhesion Measurements

**Figure 1:**
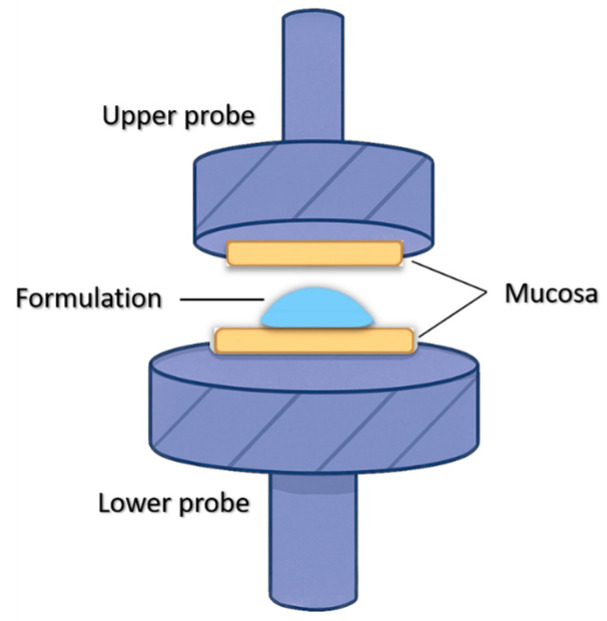
Schematic of the experimental setup for ex vivo mucoadhesion measurements using porcine buccal mucosa and texture analyzer.

Mucoadhesive strength was analyzed using a TA.XT Plus Texture Analyzer (Stable Micro Systems Ltd., Godalming, UK). Fresh porcine buccal mucosa (thickness ~2 mm; area ~1 cm^2^) was affixed to both the probe and base platform using LOCTITE^®^ Super Glue, as shown in Figure 1. A 100 μL aliquot of each formulation was applied to the mucosa surface on the base. The probe was lowered at 0.1 mm/s and a contact force of 0.5 Newton was applied and maintained for 60 s. The probe was then withdrawn at the same rate, and the peak detachment force was recorded as the mucoadhesive strength.

### 2.7. Statistical Analysis

Group differences were assessed using one-way analysis of variance (ANOVA), followed by Tukey’s post hoc test for pairwise multiple comparisons.

Correlations between formulation parameters (i.e., viscosity, mucoadhesion), in vivo residence and immunogenicity metrics (IgG and IgA titres) were evaluated using linear regression analysis. Pearson correlation coefficients (r) were computed to determine the strength and direction of associations between respective XY variable pairs. Datasets were log-transformed prior to analysis to stabilize distribution and variance, in accordance with the normality assumptions of Pearson’s test. Effect sizes were interpreted adopting Cohen’s [45] conventional thresholds for Pearson’s r corresponding to:
**ρ (Pearson’s r)****Effect Size****Interpretation**
0.10–0.29
SmallWeak correlation
0.30–0.49
MediumModerate correlation
≥0.50
LargeStrong correlation
For correlations analyses, effect sizes were prioritized in interpretation, with *p*-values reported for completeness.

All statistical analyses were performed using GraphPad Prism (version 8.4.2) for Windows (GraphPad Software, San Diego, CA, USA). Statistical significance was defined as *p* ≤ 0.05, with thresholds indicated by asterisks and specified as follows:
***p*-Value****Asterisks****Interpretation***p*
≤ 0.05
*Significant*p*
≤ 0.01
**Very significant*p*
≤ 0.001
***Highly significant*p*
≤ 0.0001
****Extremely significant

Given the exploratory nature of multifactorial correlation analyses, a relaxed significance threshold (*p* < 0.1), above the conventional *p* ≤ 0.05, was considered indicative of a meaningful trend when supported by a large effect size, and was marked with # to distinguish from conventionally significant findings.

## 3. Results

**Figure 2:**
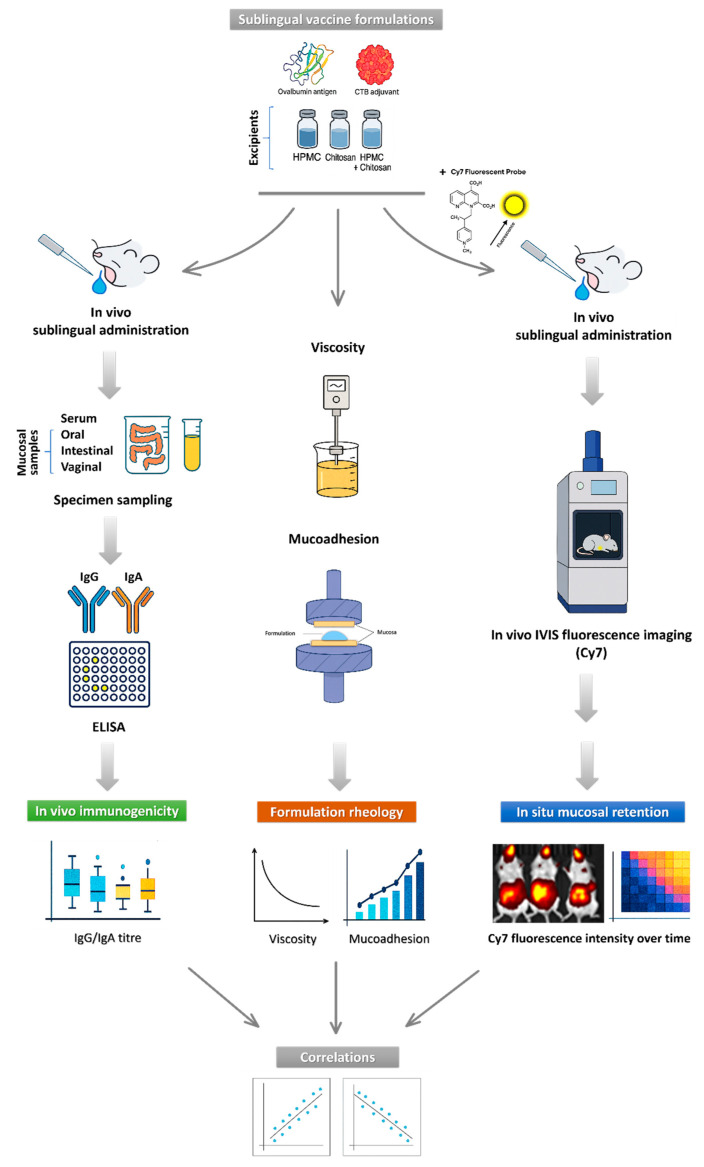
Workflow of study experimental design and analyses. The schematic illustrates the sequential steps of the study, including formulation of sublingual vaccines, which are then used for (i) sublingual immunization in mice, followed by quantification of systemic (serum IgG) and mucosal (oral, intestinal, and vaginal IgA) immune responses; (ii) in vivo IVIS imaging of sublingual vaccine in situ residence and clearance using Cy7-labeled formulations; (iii) rheological characterization, including viscosity and ex vivo mucoadhesion measurements. The study was concluded with correlation analyses linking formulation rheology, mucosal retention, and in vivo immunogenicity outcomes.

To investigate the impact of formulation characteristics on sublingual vaccine performance, a systematic experimental approach was applied. Vaccine formulations with varying combinations of antigen, adjuvant, and selected mucoadhesive excipients were administered sublingually in mice and assessed for: (i) in vivo immunogenicity profiles, including both systemic and mucosal humoral immunity, and (ii) in situ retention. Additionally, (iii) rheological analyses of vaccine formulations were conducted to measure their viscosity and mucoadhesive strength. Finally, correlation analyses were performed to integrate the above datasets and identify key relationships between vaccine formulation rheology, mucosal residence, and generated immune responses—within the sublingual context. The experimental design of our study is depicted in Figure 2.

### 3.1. Mucoadhesive Excipients as Key Modulators of Immunogenicity in Sublingual Vaccines

We screened a panel of seven vaccine formulations with different combinations of OVA antigen, CTB adjuvant, and excipients such as chitosan, and HPMC, to examine most optimal components and their configurations to induce in vivo systemic and mucosal immunity in a mouse model upon sublingual administration.

**Figure 3:**
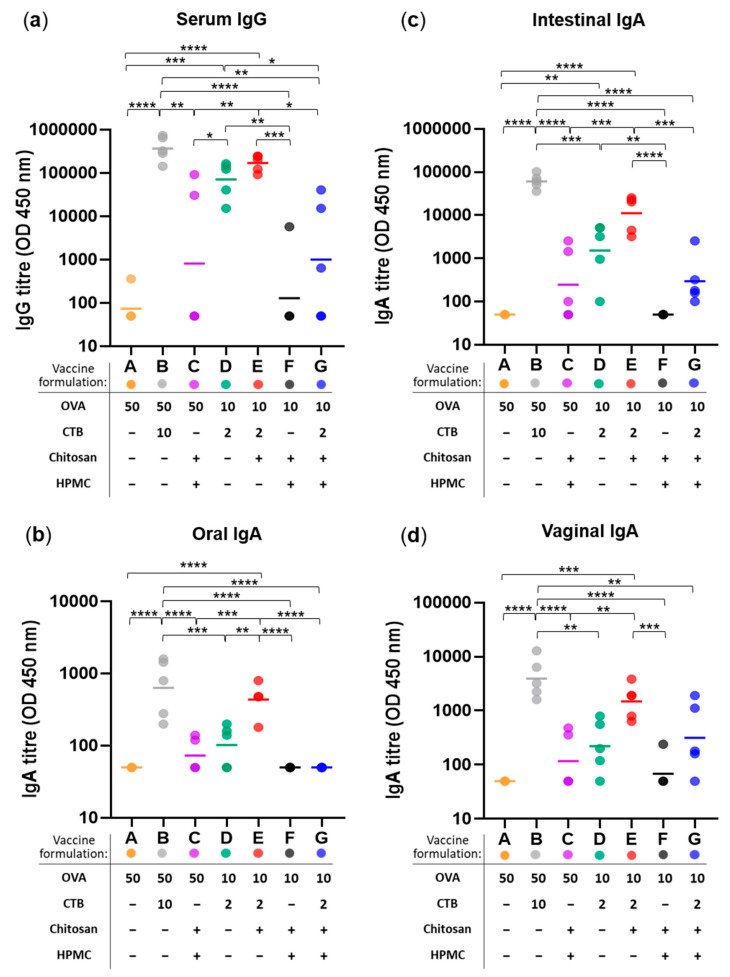
Immunogenicity of sublingual vaccine formulations measured as anti-OVA antibody responses at systemic and mucosal sites. (**a**) Serum IgG titres, (**b**) oral IgA titres, (**c**) intestinal IgA titres, and (**d**) vaginal IgA titres measured following weekly sublingual administration (over five weeks) with 7 vaccine formulations (A–G): varying in their content of antigen (OVA; at 50 or 10 µg per dose), adjuvant (CTB; at 10 or 2 µg per dose), and excipients HPMC (3% *w*/*v*) and/or chitosan (25 µg per dose)—as specified in the corresponding figure legends. Colored dots represent individual animals; horizontal bars indicate the geometric mean titre (GMT) of each group (n = 5) on log-scale. Statistical significance thresholds are indicated as follows: * *p* ≤ 0.05, ** *p* ≤ 0.01, *** *p* ≤ 0.001, **** *p* ≤ 0.0001.

In line with our previous work [35,43], weekly sublingual immunization over a five-week period using ovalbumin (OVA) as a model antigen and Cholera Toxin B subunit (CTB) as a mucosal adjuvant (Formulation B: 50 µg OVA + 10 µg CTB) induced both systemic and mucosal humoral responses, including the highest serum IgG and IgA antibody titers across oral, intestinal, and vaginal mucosa—thus serving as the positive control (Figure 3). The selected concentrations of OVA and CTB were guided by our earlier dose–response studies and align with published literature demonstrating their immunogenicity in mucosal vaccine platforms [35,41,46,47,48]. Reduced antigen and adjuvant doses (Formulation D: 10 µg OVA + 2 µg CTB) significantly lowered serum IgG (Figure 3a) and IgA titers at all mucosal sites (Figure 3b–d). Adjuvant-free formulation (Formulation A) generated minimal immune responses and served as the negative control.

Additionally, HPMC and chitosan (MGC) were incorporated into the vaccine formulations as excipients enhancing viscosity, mucoadhesion, and/or permeation—the properties perceived to benefit sublingual delivery due to extending antigen contact time and uptake. Their respective concentrations were selected based on previously published studies demonstrating optimal rheological or immunomodulatory performance in mucosal delivery systems [21,37,38,49,50].

The addition of chitosan (Formulation E) to low-dose OVA + CTB control (Formulation D) increased both serum IgG and mucosal IgA to levels comparable to the high-dose positive control (Formulation B), with statistical significance reached for oral IgA relative to the control (** *p* < 0.01)—indicating chitosan’s antigen and adjuvant dose-sparing effect.

However, co-formulation with film-forming HPMC (Formulation G) consistently resulted in reduced antibody titers—including serum IgG (169-fold decrease relative to Formulation E, * *p* < 0.05) and mucosal IgA in the intestinal (37-fold decrease vs. E, *** *p* < 0.001), oral (9-fold decrease vs. E, **** *p* < 0.0001), and vaginal (5-fold decrease vs. E, p > 0.05) sites. The rationale for using HPMC in sublingual vaccine formulations – a well-established viscosity enhancer [42,43] – was to produce a more viscous formulation matrix that would remain in situ and minimize its rapid drainage into the stomach. It was hypothesized that the presence of HPMC and prolonged contact time in the sublingual space would enhance immune responses, yet our findings revealed the opposite—controversially suggesting its rather inhibitory effect on sublingual immunization.

### 3.2. Impact of Mucoadhesive Excipients on In Situ Mucosal Retention of Sublingual Vaccines

To further investigate the unexpected reduction in immunogenicity associated with HPMC, in vivo residence and biodistribution were evaluated over a 28-h period using Cy7-labeled formulations tracked via live IVIS imaging, following sublingual administration in mice.

**Figure 4:**
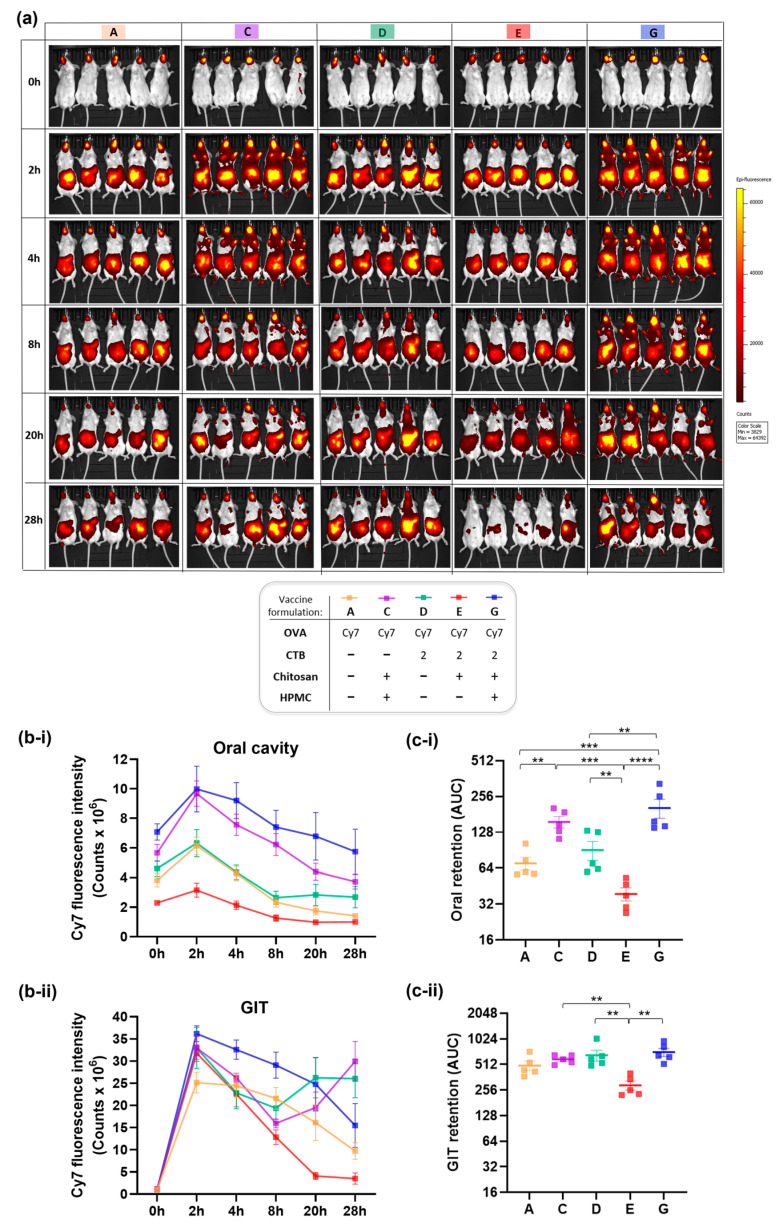
In vivo residence and fate of sublingually administered vaccine formulations labeled with Cy7 fluorescent probe and monitored using IVIS fluorescence imaging in mice. (**a**) Representative IVIS images illustrating spatiotemporal biodistribution and clearance dynamics of Cy7 signal in mice over time (0–28 h), visualized as heatmaps. (**b**) Quantification of Cy7 fluorescence as epifluorescence counts, plotted as linear graphs to represent kinetics over time, showing: (**b-i**) Cy7 fluorescence intensity in the oral cavity, and (**b-ii**) in the gastrointestinal tract (GIT). (**c**) Total retention per each vaccine formulation was calculated as area under the curve (AUC) using trapezoidal integration analysis, and shown on log-scale for (**c-i**) oral cavity and (**c-ii**) GIT. Data are presented as mean ± SEM (n = 5 per group). Vaccine formulations analyzed: A, C-E and G (formulation content and corresponding color-coded identifiers are provided in the figure legend). Statistical significance thresholds are indicated as follows: ** *p* ≤ 0.01, *** *p* ≤ 0.001, **** *p* ≤ 0.0001.

Figure 4a,b show Cy7 fluorescence intensity in the oral cavity versus gastrointestinal tract (GIT), measured at multiple time points over 28 h to capture the clearance kinetics. GIT represents one potential route of formulation drainage, driven by salivary flow and the swallowing reflex. Alternative fates for sublingual vaccine formulations include mucosal absorption and subsequent entry into systemic or lymphatic circulation [1,4].

As shown in Figure 4b-i, Cy7 fluorescence signal was detected in the oral cavity for all formulations, peaking at t = 2 h post sublingual administration, which aligns with previous reports studying mucoadhesive polymers, including chitosan [51,52]. The signal gradually declined over time, reaching its lowest levels at the terminal time point of t = 28 h. Interestingly, the fluorescence intensity varied across formulations from the outset, with the differences particularly evident in those containing chitosan as standalone excipient (Formulation E), or supplemented with HPMC (Formulations C and G)—corresponding to lower and higher signal compared to excipient-free controls, respectively. 

At 2 h post-sublingual administration, the results consistently showed lower Cy7 signal in the oral cavity for the chitosan-based formulation E compared to the HPMC-containing formulations C and G. This suggests that the Cy7 tracer could be more readily absorbed in this group—an outcome consistent with chitosan’s established permeation-enhancing capabilities and the rich vascular and lymphatic architecture of the sublingual space. Following mucosal absorption, the tracer may have entered systemic circulation or been trafficked via lymphatic drainage pathways, resulting in reduced local Cy7 signal intensity in these mice. On the contrary, HPMC-containing vaccine formulations exhibited the highest Cy7 fluorescence in the oral cavity—appearing to limit such mucosal absorption even despite the co-presence of chitosan (Formulations C and G). Therefore, based on Cy7 fluorescence levels in the oral cavity at 2 h post-sublingual administration, the formulation-dependent kinetics of mucosal absorption for sublingual vaccines could be extrapolated as follows: chitosan > excipient-free > HPMC in a descending order from most to least efficient.

The same trend remained consistent for later timepoints: oral residence varied by formulation, with HPMC-containing gels (Formulations G and C) exhibiting significantly greater retention relative to non-HPMC counterparts (Formulations E, A, and D). Among the latter, the chitosan-containing formulation E drained most rapidly, which aligns with its permeation-enhancing properties. Remarkably, when chitosan was co-formulated with HPMC (Formulations G and C), its permeation enhancer effect was suppressed, while the HPMC’s retentive capacity remained fully preserved. This observation closely mirrors the immunological profiles we earlier described for the same formulations—where chitosan alone boosted immune responses, while its immunogenic potency was compromised in the presence of HPMC. Further evidence of slower drainage—and consequently prolonged oral residence—was demonstrated exclusively in mice given HPMC-containing formulations (C and G) by Cy7 fluorescence detected on their front paws. This observation suggests grooming-related transfer, potentially triggered by discomfort or irritation associated with the dense, gel-like consistency of the HPMC matrix.

In the gastrointestinal tract (Figure 4b-ii), no signal was detected at t = 0 h, as expected. The fluorescence intensity peaked at t = 2 h post-sublingual administration, then gradually decreased over time for all formulations except D and C, which recorded unexplained second peaks at t = 20 h and terminal t = 28 h, respectively. The peak gastrointestinal signal observed at 2 h across all formulations may reflect the time required for partial clearance from the oral cavity, facilitated by salivation and swallowing. Overall, consistent with the oral cavity kinetics, HPMC-containing formulations (G and C) exhibited the slowest clearance also in the gastrointestinal tract, whereas chitosan (Formulation E) showed the most accelerated drainage profile.

Finally, total quantifiable retention for each formulation was computed as the area under the curve (AUC) from Cy7 distribution kinetics profiles, providing a comparative measure of mucosal residence across vaccine formulations (Figure 4c). It confirmed a significant 2.3-fold reduction (** *p* < 0.01) in oral retention (Figure 4c-i) for the chitosan-based formulation free of HPMC (Formulation E), while significant increase (also by 2.3-fold, ** *p* < 0.01) was demonstrated for the HPMC-containing counterpart (Formulation G), relative to the excipient-free control (Formulation D). A similar formulation-dependent trend was observed for total retention in the gastrointestinal tract (Figure 4c-ii). This ultimately supports the previously described patterns of accelerated absorption driven by the permeation-enhancing properties of chitosan, and prolonged mucosal retention with delayed drainage for the HPMC-containing formulations—likely attributable to HPMC’s high viscosity and film-forming capacity.

### 3.3. Rheological Insights into Mucosal Retention of Sublingual Vaccine Formulations 

**Figure 5:**
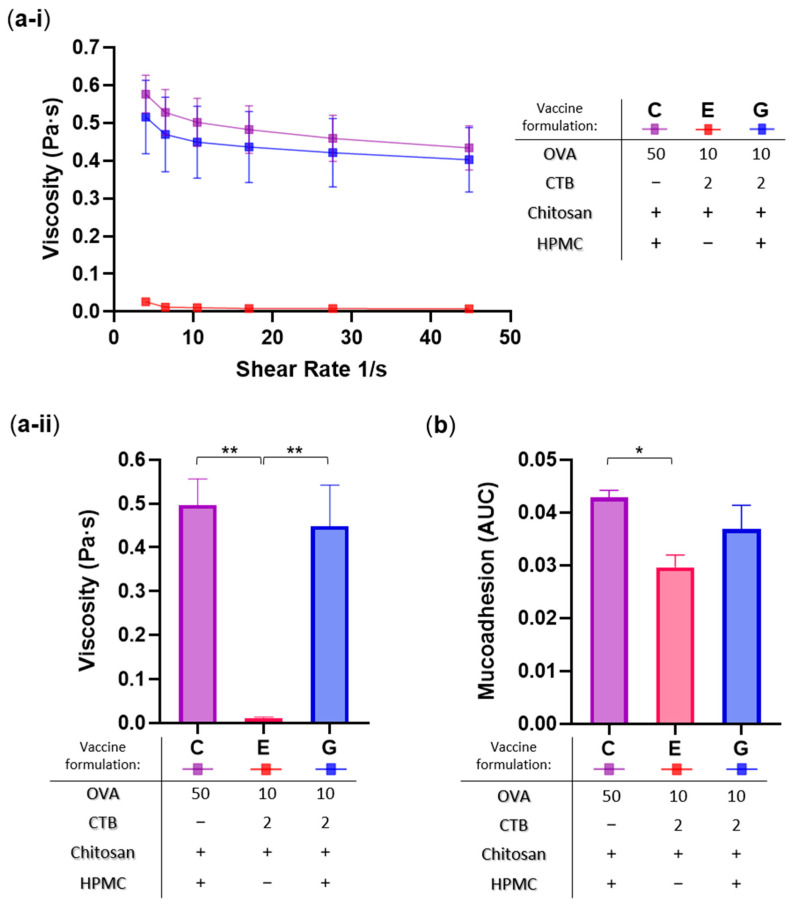
Rheological characterization of selected sublingual vaccine formulations. Formulations analyzed: C (50 µg OVA + 25 µg chitosan + 3% *w/v* HPMC), E (10 µg OVA + 2 µg CTB + 25 µg chitosan), and G (10 µg OVA + 2 µg CTB + 25 µg chitosan + 3% *w/v* HPMC). (**a**) Viscosity profiles: (**a-i**) measured across varying shear rates, and (**a-ii**) total averages presented as bar graph for direct comparison and statistical analysis. (**b**) Mucoadhesion assessed by tensile force analysis, and expressed as area under the curve (AUC). Data are presented as mean ± SEM (n = 3); statistical significance thresholds are indicated as follows: * *p* ≤ 0.05, ** *p* ≤ 0.01.

Next, to evaluate the influence of viscosity and mucoadhesion on sublingual retention, vaccine formulations containing mucoadhesive excipients and exhibiting the highest (Formulations C and G) and lowest (Formulation E) oral residence were subjected to rheological characterization (Figure 5).

Viscosity was measured at different shear rates (Figure 5a-i) and the average values were computed for statistical analysis (Figure 5a-ii), while mucoadhesion was quantified as tensile force and expressed as the Area Under the Curve (AUC) using porcine mucosa as the model (Figure 5b). As expected, vaccine formulations containing HPMC (C and G) demonstrated significantly higher viscosity (42- and 38-fold, respectively; ** *p* < 0.01), and moderately increased mucoadhesive strength (1.5-fold, * *p* < 0.05; and 1.3-fold, *p* > 0.05, respectively), compared to the HPMC-free chitosan (Formulation E). These results are consistent with the established role of HPMC as a gelling agent in pharmaceutical formulations. Furthermore, they also closely align with the in situ oral residence patterns shown above, highlighting the direct influence of formulation rheology on sublingual retention.

### 3.4. Correlation Analyses Uncover Key “Drivers” and “Brakes” of Sublingual Vaccine Efficacy

Furthermore, comparison of formulations’ rheological properties with their respective immunogenicity outcomes revealed a paradoxical trend in sublingual immunization: the most viscous and mucoadhesive formulations (C and G) elicited the weakest immune responses, whereas the low-viscosity and less mucoadhesive formulation E induced markedly stronger systemic and mucosal immunity.

**Figure 6:**
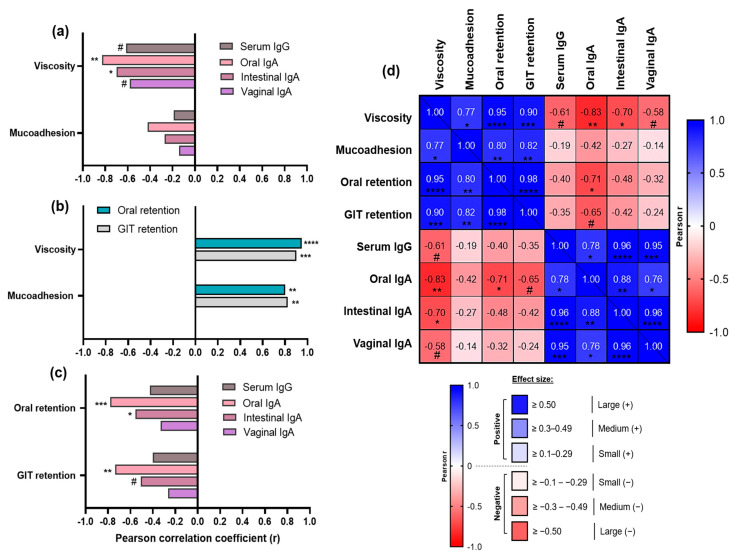
Correlation analyses between rheological properties of sublingual vaccine formulations containing mucoadhesive excipients (C, E and G), their in situ/ex situ mucosal residence, and in vivo immunogenicity outcomes. (**a**) Relationship between rheological parameters (viscosity and mucoadhesion), and immunogenicity markers (serum IgG and mucosal IgA responses). (**b**) Relationship between rheological parameters and oral/gastrointestinal (GIT) retention expressed in AUC. (**c**) Relationship between oral/GIT retention and immunogenicity markers. For panels (**a**–**c**) Pearson correlation coefficients (r) are plotted on the *x*-axis as bars for visual comparison, with positive or negative values indicating the direction of correlation. (**d**) Multifactorial correlation matrix heatmap summarizing relationships across all tested parameters: encoding both correlation direction (blue for positive, red for negative) and magnitude (color intensity gradient proportional to effect size) based on Pearson r values, and with corresponding statistical significance levels denoted. Sample size ranges: n = 9–15 XY variable pairs. Statistical significance thresholds are indicated as follows: * *p* ≤ 0.05, ** *p* ≤ 0.01, *** *p* ≤ 0.001, **** *p* ≤ 0.0001; exploratory trends approaching conventional significance but *p* > 0.05 are denoted by # (*p* < 0.1).

Correlation analyses depicted in Figure 6 substantiated these findings, revealing two key groups of associations for vaccine formulations containing mucoadhesive excipients in the context of sublingual delivery. Firstly, strong positive correlations were observed between formulation rheological characteristics (viscosity, mucoadhesion) and their in situ/ex situ retention, suggesting these parameters are tightly interdependent and confirming that more viscous and mucoadhesive formulations persisted more readily at the sublingual administration site (Figure 6b). Notably, viscosity correlated strongly with oral (r = 0.95, **** *p* < 0.0001) and GIT retention (r = 0.90, *** *p* < 0.001). Mucoadhesion also showed significant positive associations with the same residence metrics, albeit to a comparatively lesser extent (r = 0.80 and 0.82, respectively, ** *p* < 0.01).

Secondly, distinct inverse correlations emerged between formulation rheological properties (Figure 6a), alongside their oral/GIT retention (Figure 6c), and the magnitudes of in vivo immune responses they generated. Specifically, among the rheological parameters, viscosity exhibited the strongest negative associations with immunogenicity outcomes (Figure 6a), including oral IgA (r = −0.83, ** *p* < 0.01) and intestinal IgA (r = −0.70, * *p* < 0.05), and to a lesser extent with serum IgG and vaginal IgA—which, despite not reaching conventional statistical significance thresholds (*p* < 0.1), may nonetheless reflect a meaningful trend for biological relevance due to their large effect sizes (r = −0.61 and –0.58, respectively). On the other hand, mucoadhesion demonstrated only weak (with serum IgG, intestinal IgA, and vaginal IgA) to moderate (oral IgA) negative relationships with the immunogenicity markers. 

Likewise, mucosal residence metrics—particularly oral retention—were negatively correlated with mucosal IgA levels, showing potent and statistically significant associations for oral IgA (r = −0.78, *** *p* < 0.001) and intestinal IgA (r = −0.55, * *p* < 0.05), with the exception of vaginal IgA, where the negative correlation was moderate (r = −0.33) and did not reach statistical significance. Similar trends were observed for the GIT retention.

Additionally, we observed in our dataset that the inverse correlation between oral residence and immune responses appears strongest at early timepoints (0–4 h) and incrementally weakens as time progresses (20–28 h). This temporal dynamics pattern was found highly consistent and suggests the existence of a time-sensitive window for optimal mucosal stimulation in the sublingual context. The initial vaccine contact time may play a vital role in antigen uptake and immune priming. Over time, salivary clearance mechanisms are more likely to cause vaccine drainage and reduce antigen availability and, consequently, its immunogenic impact.

Furthermore, a multifactorial correlation matrix heatmap was generated across all experimental readouts (Figure 6d), to summarize formulation-level relationships among physicochemical parameters (viscosity, mucoadhesion), in situ/ex situ residence metrics (oral and gastrointestinal retention), and in vivo immunogenicity outcomes (serum IgG, oral, intestinal, and vaginal IgA titres). These correlations recapitulated the previously highlighted associations, but also added further depth and context to the observed relationships. For example, it demonstrated that mucosal antibody responses were strongly interrelated. Local oral IgA production showed the strongest positive correlation with intestinal IgA (r = 0.88, ** *p* < 0.01), followed by vaginal IgA (r = 0.76, * *p* < 0.05), among distal mucosal sites—highlighting coordinated IgA induction across multiple mucosal compartments, yet perhaps preferential activation of gut-associated lymphoid tissues (GALT) over vulvovaginal-associated lymphoid tissues (VALT). Interestingly, based on effect size, serum IgG was most strongly correlated with intestinal IgA (r = 0.96, **** *p* < 0.0001), followed by vaginal IgA (r = 0.95, *** *p* < 0.001), and oral IgA (r = 0.78, * *p* < 0.05). These potent positive associations indicate substantial overlap between systemic and mucosal immune responses, further supporting a prominent role for GALT among all mucosa-associated lymphoid tissue (MALT) compartments in engaging not only mucosal but also systemic immunity. This is likely underpinned by enhanced drainage pathways to GIT, as well as GALT’s anatomical advantages, including highest mucosal surface area and rich vasculature.

Taken together, these findings demonstrate that while formulation properties such as viscosity and its mucosal retention are tightly interdependent, they are inversely associated with both systemic and mucosal antibody responses. It appears that even subtle differences in antigen mobility across viscous and/or mucoadhesive formulation matrices in the sublingual context may contribute to gradations in immune response profiles—reflecting both locally and across distal mucosal compartments, given the interconnected nature of mucosal immunity. 

## 4. Discussion

This study systematically investigated the interplay between sublingual vaccine formulation rheology, in situ mucosal residence, and in vivo immunogenicity in a murine model, revealing unexpected yet consistent patterns of immune engagement.

Although mucoadhesive excipients are often pursued to extend residence and protect antigen from clearance, our results demonstrate that they may in fact impair immune responses. Higher viscosity and stronger mucoadhesion of hydroxypropyl methylcellulose (HPMC)-based formulations—while mediating prolonged sublingual retention—produced weaker systemic and mucosal antibody responses. These results suggest that HPMC, despite its widespread use as a viscosity enhancer favoring mucosal contact time, may be suboptimal for sublingual vaccine delivery. In contrast, less viscous and less mucoadhesive chitosan formulation boosted immune responses, even when antigen and adjuvant doses were reduced. Below we interpret these observations, reconcile them with prior literature, and discuss likely mechanisms and implications for sublingual vaccine design.

### 4.1. Sublingual Immunization: Impact of Vaccine Formulation on Efficacy and Its Mechanistic Insights 

Sublingual delivery is an attractive mucosal vaccination route as it combines needle-free administration, ability to induce both systemic and mucosal immune responses, while avoiding the gastrointestinal lumen and first-pass hepatic metabolism [2,4,27,53,54]. 

Our findings that sublingual vaccine formulations are able to elicit strong serum IgG and broad mucosal IgA responses—not only at the local oral site but also at distal mucosal compartments (intestinal, vaginal)—are consistent with prior reports demonstrating that sublingual delivery can prime both systemic and mucosal immunity [3,4,53]. This capacity depends critically on antigen availability to antigen-presenting cells (APCs) in the sublingual mucosa and to downstream induction sites [55,56,57], and is further supported by adjuvants to override the inherently tolerogenic bias of mucosal tissues. CTB is a well-established mucosal adjuvant that has been used with a broad range of antigens at various doses, and has shown a dose-dependent immunostimulatory effect [40,41,46,58,59,60].

To place it in context and enable more comprehensive understanding, it is essential to consider the anatomical and physiological characteristics of the sublingual mucosa, which may influence antigen uptake, distribution, and subsequent immune engagement. The sublingual mucosa is thin and highly vascularized, enabling antigens and other substances or particles to: (i) traverse the epithelial barrier via transcellular or paracellular routes and penetrate into the sub-epithelial tissue, where APCs reside and can internalize and process antigens for presentation to T cells, thereby initiating local immune responses. Alternatively, (ii) antigens may be absorbed into local blood capillaries, further to lingual and sublingual veins draining into the internal jugular vein, to subsequently enter systemic circulation; or (iii) lymphatic vessels—leading to drainage into regional lymph nodes. Specifically, the sublingual and submandibular regions drain into the deep cervical lymph nodes, which are part of the broader mucosa-associated lymphoid tissue (MALT) system—a key network for immune activation. Moreover, antigen-bearing dendritic cells or Langerhans cells originating from the sublingual mucosa may migrate to distant lymphoid organs, trafficking antigen and contributing to the priming of mucosal immunity at remote sites, such as gut-associated lymphoid tissue (GALT) [1,4]. Therefore, the overall fate of the sublingual vaccine—and the resulting immunological outcomes – are likely governed by the dynamic interplay between mucosal retention and uptake, local immune processing, systemic distribution, and gastrointestinal clearance.

Mucoadhesive excipients are traditionally employed to prolong mucosal residence and reduce the rapid clearance of antigens caused by salivation, swallowing, or enzymatic degradation. Consequently, it is widely assumed that extended mucosal contact should promote antigen exposure to APCs, thereby facilitating immune recognition and processing [61]. Based on this rationale, the hypothesis underlying the inclusion of HPMC in sublingual vaccine formulations—that enhanced viscosity and mucoadhesion would prolong mucosal contact, shield the antigen from salivary clearance/enzymatic degradation, and thereby improve its uptake and induction of immune responses—was mechanistically grounded.

However, our data challenge this paradigm, as formulations containing HPMC consistently suppressed immunogenicity compared with HPMC-free counterparts. HPMC-containing gels exhibited the highest viscosity and mucoadhesion, along with strongest in situ retention, delaying clearance from the oral cavity. However, instead of boosting immunity, HPMC-formulated vaccines produced the lowest serum IgG and mucosal IgA titers, even when CTB was included as a potent mucosal adjuvant. Our findings are supported by prior work by Wheeler et al., who reported that Carbopol—another commonly used mucoadhesive polymer and gelling agent—reduced the immunogenicity of sublingually administered vaccines in mice, whereas Carbopol-free aqueous formulations elicited stronger antibody responses [62]. Likewise, studies on nasal and buccal delivery systems have indicated that excessive viscosity and strong mucoadhesion can hinder the timely release of payloads—including antigens—and restrict diffusion to epithelial surfaces, thereby limiting access to APCs [3,51,52].

### 4.2. HPMC: Immunological Trade-Offs of High Rheology and In Situ Retention

We considered three plausible mechanisms that could potentially explain this paradox and link HPMC’s high rheology to reduced immunogenicity: (i) restricted antigen release, (ii) lack of productive APC engagement, and (iii) diversion toward clearance and tolerogenic pathways. 

HPMC is a widely used pharmaceutical gelling agent—enhancing viscosity and mucoadhesion [37,38]. It exists in multiple grades with differing methoxy and hydroxypropoxy substitution and molecular weight. Gustafsson et al. and recent reviews have shown that substitution pattern alters HPMC gel strength, hydration and payload release behavior. The unmodified HPMC used in our study exhibits 29% methoxy, 10% hydroxypropoxy substitution. More highly methoxylated variants tend to form stronger hydrophobic interactions, enhancing compaction and viscosity, retarding solubilization, and delaying release of incorporated payloads [63,64,65,66,67,68,69]. Consequently, the physical entrapment of antigens within HPMC’s dense gel-like matrix likely limits antigen diffusion and its subsequent transport across the mucosal epithelium—restricting its bioavailability for uptake by dendritic cells and Langerhans cells in the sublingual mucosa, thereby tempering effective immune priming [38,70,71].

Finally, prolonged antigen presence in the oral cavity increases the chance of it being swallowed and processed via the gastrointestinal tract or presented in a tolerogenic context. The mucosal immune system is designed to maintain tolerance to food and commensal antigens, and repeated exposure without danger signals can activate regulatory pathways dampening systemic and mucosal immune responses. Mechanistic studies show that sustained mucosal antigen exposure, especially without strong pro-inflammatory adjuvants, recruits tolerogenic dendritic cells and regulatory T cells [72,73,74]. Tolerogenic pathways may help explain why our HPMC-based formulations with CTB were more immunopotent than their counterparts lacking CTB, yet they do not account for the broader suppression of immune responses shown by the adjuvant-containing HPMC variant relative to excipient-free controls. Although CTB—a potent mucosal adjuvant [40,41,46]—was incorporated into select HPMC-based formulations, its likely co-entrapment with the antigen within the dense gel matrix may have hindered exposure to sub-mucosal APCs, ultimately failing to elicit the desired immune responses. Therefore, although HPMC enhances mucosal retention—thus prolonging antigen dwell time at the sublingual site—its failure to permit efficient physical access to relevant immune processing may paradoxically shift the response toward tolerogenic rather than immunostimulatory priming. 

It is important to emphasize that this retarded macromolecule diffusion trait and HPMC hydrogel depot strategies are still advantageous for sustained drug delivery and capable of upregulating immune responses in other anatomical routes and contexts, such as injectable subcutaneous depots. Gale et al. and Ou et al. demonstrated that a subcutaneous depot of injectable HPMC-based hydrogel delivering the SARS-CoV-2 RBD antigen produced robust humoral immunity in mice [75,76]. However, this efficacy was likely supported by antigen presentation within the adjuvant-rich, pro-inflammatory microenvironment of the subcutaneous compartment—immunologically conducive to productive immune priming [77]. Such context biologically contrasts the thin, highly secretory sublingual mucosa where salivary flow, epithelial tight junctions, and local tolerogenic mechanisms dominate. This key distinction helps explain why hydrogel depots can be immunostimulatory when administered subcutaneously, yet counterproductive as highly retentive antigen delivery platform at the sublingual mucosa. 

Indeed, studies of controlled-release tablet vaccines in the sublingual context have shown that more rapid antigen release correlates with stronger humoral responses, while extended release formulations tend to be less immunogenic, likely due to limited antigen bioavailability at the mucosal epithelium [32,78,79]. These observations align with the present data showing greater responses from faster-releasing formulations. 

Our findings are thus in line with the emerging consensus that materials should be tuned to the route’s rate-limiting step. Depot-forming, highly retentive matrices suit subcutaneous delivery, whereas sublingual vaccination may favor less viscous, mucus-penetrating and permeation-enhancing systems. 

### 4.3. Chitosan: Balancing Permeation and Adjuvanticity for Optimal Sublingual Vaccine Performance

The latter characteristic would be consistent with chitosan’s permeation enhancer activity [80,81,82,83]. Indeed, in contrast to HPMC, chitosan-based formulations demonstrated improved immunogenicity profile despite faster clearance from the oral cavity. Chitosan’s cationic primary amines interact electrostatically with negatively charged mucins and epithelial surfaces, and have been shown to transiently open epithelial tight junctions—enhancing paracellular transport of macromolecules. This capability may facilitate antigen absorption, increasing its bioavailability to subepithelial APCs [21,43,80]. 

Independent of this permeation effect, chitosan and chitosan-based nanoparticles exhibit intrinsic adjuvant properties, including APC stimulation via innate immune pathways and promoting antigen uptake and processing [21,43,84,85,86].

Collectively, both these features likely underlie the chitosan-induced immunopotentiation observed in our study. In line with our findings, Spinner et al. also reported enhanced mucosal and systemic responses in mice at concentrations of 5–25 µg – consistent with our 25 µg dose [21]. Our work, therefore, contributes to the growing body of evidence supporting chitosan’s dual role as a multifunctional mucosal delivery enhancer and adjuvant platform [85,87]. 

We even observed a dose-sparing benefit of incorporating chitosan as an excipient in sublingual vaccine formulations—capable of compensating for reduced antigen and adjuvant concentrations in terms of boosting immunity. Mechanistically, this dose-sparing effect may be attributed to chitosan’s capacity to enhance paracellular permeability, thereby increasing the fraction of antigen and adjuvant that comes into contact with dendritic cells or undergoes transcytosis to draining lymph nodes. This ultimately amplifies immune priming despite a lower initial antigen/adjuvant load at the mucosal surface. 

Interestingly, when HPMC was combined with chitosan, the beneficial effects of chitosan were lost, and immune responses were suppressed. This suggests that HPMC not only restrains antigen release but may also interfere with chitosan’s permeation-enhancing capacity, possibly by limiting its access to epithelial tight junctions. This antagonistic interaction underscores the importance of excipient–excipient compatibility in mucosal vaccine design. HPMC could be suitable for applications where masking chitosan’s immunogenic effects is desirable, but appears suboptimal in vaccine formulations where immunization is the primary objective—specifically in the sublingual context.

### 4.4. Strengths, Limitations, and Future Work

A major strength of this study lies in its integrated, multi-modal approach, which combines and correlates: (i) characterization of vaccine formulation rheological properties, including viscosity and mucoadhesion; (ii) quantitative in vivo imaging of their in situ/ex situ retention; and (iii) in vivo immunogenicity readouts across systemic and mucosal compartments. Collectively, these complementary modalities were designed to further the understanding of formulation-dependent mechanisms governing vaccine efficacy in the sublingual delivery context, and ultimately contribute to refining their design stra-tegies.

Nonetheless, several limitations should be acknowledged. First, in vivo residence was inferred from Cy7 fluorescence tracer distribution rather than labeled antigen. While common practice [88,89], dye diffusion/absorption kinetics can differ from those of protein antigens and therefore may not perfectly reflect antigen bioavailability. We agree that labeling the antigen itself (i.e., Cy7-conjugated OVA) could provide more direct antigen biodistribution data. However, this approach presents several limitations in the context of our study: (i) conjugation process may alter antigen structure and uptake behavior—Cy7 conjugation can increase the hydrodynamic radius of OVA, potentially reducing mucosal permeability and inadvertently perturbing its native transport kinetics nonetheless; (ii) signal variability due to labeling efficiency and protein-conjugated Cy7 quenching/reduced photostability could distort signal intensity and compromise quantitative comparisons. OVA contains fewer lysine residues as conjugation sites than, for example, antibodies as typical conjugation targets and not all are solvent-accessible, limiting conjugation efficiency and increasing the risk of structural disruption. Labeling reactions often yield heterogeneous products with variable dye-to-protein ratios, introducing batch-to-batch inconsistency and complicating interpretation. Limited yield of Cy7 as OVA-conjugated fraction could lower the signal intensity for robust in vivo detection and quantification in live animals by IVIS. Additionally, (iii) Cy7–OVA conjugates may undergo partial deconjugation or shedding in vivo. The amide bond formed during NHS ester conjugation is susceptible to hydrolysis under physiological conditions, and proteolytic cleavage of OVA in mucosal tissues may release Cy7-labeled fragments or free dye. This could compromise the accuracy of biodistribution tracking, as the fluorescent signal may no longer reflect intact antigen behavior [90,91,92,93,94]. These factors collectively reinforced our decision to use free dye as a simplified but more reliable surrogate for formulation-wide retention tracking. The use of free Cy7 dye provided a consistent and reproducible signal across formulations, enabling comparative analysis of excipient-driven retention kinetics in quantitative terms. However, future studies incorporating labeled antigens in a stable and optimal manner would complement our findings and help delineate the relationship between formulation retention and antigen translocation. 

Secondly, for practical reasons ex vivo mucoadhesion experiments were conducted using porcine buccal mucosa—a widely accepted surrogate for human oral mucosa due to its morphological similarity [95,96]. However, interspecies differences relative to murine sublingual tissue may introduce discrepancies in adhesion magnitudes, though directional trends should largely be conserved. Furthermore, we evaluated a single, unmodified HPMC (∼29% methoxy/10% hydroxypropoxy) at 3% (*w*/*v*) concentration as our model excipient for high viscosity and mucoadhesion. Perhaps lower concentrations, other substitution patterns, molecular weights, or polymer blends might reduce diffusional barriers and perform differently—which presents an opportunity for future optimization. We have noted that hydrogels were able to induce immune responses in other more immunologically primed contexts, such as subcutaneous depot administration [75,76,77]. Direct route-specific comparisons of our vaccine formulation containing 3% HPMC between sublingual and other delivery routes, e.g., subcutaneous and oral, would be valuable to ultimately determine whether its observed immune profiles are specifically attributable to the sublingual route or reflect intrinsic properties of the formulation itself. 

Finally, while our findings are consistent and statistically grounded, the study did not directly measure antigen release kinetics from HPMC matrices or evaluate antigen uptake by APCs, which would strengthen the proposed mechanistic causality. Our study also focused on humoral immune responses and did not investigate cell-mediated immunity. 

Future studies should build on this foundation by: (i) incorporating broader immunological endpoints, expanding our work to include T-cell profiling and cytokine analysis to provide a more comprehensive immunological assessment, (ii) including profiling of antigen release and APC recruitment at the sublingual site, and (iii) evaluating whether HPMC grades with altered substitution patterns and/or lower viscosity can strike a better balance between mucosal retention and antigen diffusion in the sublingual context. 

### 4.5. Implications for Sublingual Vaccine Formulation Design

Our correlation analyses further reinforced the above-mentioned mechanistic interpretations, highlighting important implications for the rational design of sublingual vaccine formulations. In the sublingual vaccine context, achieving excess mucosal retention by increasing viscosity and mucoadhesion may be counterproductive without adequate antigen release and mucosal penetration to make it accessible to the APCs for immune priming, or if it promotes downstream clearance, or tolerance. While mucoadhesive polymers like HPMC are effective in prolonging antigen dwell time and protecting against enzymatic degradation, they may paradoxically impair immunogenicity by restricting antigen exposure to mucosal surfaces and their resident APCs. Conversely, formulation strategies—with chitosan being an example—that maximize antigen absorption for more effective uptake by mucosal and sub-mucosal APCs, even at the expense of reduced in situ retention, may prove more promising for sublingual vaccination. Overall, enhancing antigen diffusion and its mucosal penetration, rather than merely prolonging its dwell time, appear to be critical for preserving immunogenicity—and key determinants in the advancement of effective sublingual vaccine platforms.

Integrating our data with prior work [70,97] suggests a simplified design model for more effective sublingual vaccination: (i) high viscosity and strong mucoadhesion excipients like HPMC can reduce antigen release by immobilizing them in a dense gel-mucus network; (ii) antigens must be able to escape the formulation and traverse the mucus-epithelial barrier to access subepithelial immune cells, blood capillaries, and draining lymphatics; (iii) permeation enhancers like chitosan—by loosening epithelial junctions and facilitating translocation across the mucosal surface—are able to enhance antigen absorption, consequently improving its exposure to APCs and other immunological compartments and mechanisms—ultimately promoting effective immune stimulation. 

## 5. Conclusions

Our study elucidated the complex interplay between formulation rheology, in situ retention, and immunogenicity in the context of sublingual vaccine delivery. It offers systematically compelling evidence that increased viscosity, mucoadhesion, and oral residence—though conventionally viewed as advantageous for mucosal antigen retention—can paradoxically impair sublingual vaccine performance by adversely affecting immune responses. Sublingual vaccine formulations containing HPMC as viscosity and mucoadhesion enhancer, although persisted longer in situ in the oral cavity space, consistently elicited weaker systemic and mucosal antibody responses compared to HPMC-free counterparts. In contrast, more rapidly draining chitosan improved immunogenicity, enabling dose-sparing of both antigen and adjuvant. Correlation analyses demonstrated a clear negative relationship between formulation’s rheology/mucosal residence and immunogenicity, contradicting the prevailing assumption that prolonged sublingual retention is inherently beneficial.

Our study argues that, despite its widespread pharmaceutical application, HPMC should be used with caution for sublingual vaccination. Although it persists in situ with highest efficiency due to high viscosity and mucoadhesive properties—these same characteristics likely restrict antigen diffusion, and consequently, its availability at the mucosal–APC interface required for effective immune priming. This limited immunocompetence might be further hindered by tolerogenic exposure. Future studies should further explore the mechanistic basis of HPMC’s immunosuppressive effects in the sublingual context. Additionally, modified HPMC derivatives or alternative gelling agents with tailored antigen release profiles warrant further investigation. 

In contrast, despite the shorter antigen dwell time at the sublingual site, chitosan demonstrated superior immunization potential. This is likely attributed to its ability to enhance mucosal permeability, allowing the antigen to be more readily shuttled for uptake by the local APCs. As a result, chitosan supported more effective immune activation even at reduced antigen-adjuvant doses, revealing its dose-sparing capability in sublingual—and more broadly mucosal—vaccine development.

Overall, this work provides valuable insights into the design of sublingual vaccine formulations, emphasizing that excipient choice critically influences sublingual vaccine efficacy. While viscosity and mucoadhesion are essential for preventing rapid clearance of the vaccine from the sublingual site into the gastrointestinal tract, our findings highlight the need for a balanced formulation strategy that tailors rheological properties to not only support antigen anchoring in situ but—more importantly—prioritize its functional bioavailability to mucosal APCs by maximizing efficient antigen release and mucosal penetration. Additionally, access to sub-mucosal blood capillaries and draining lymphatics could facilitate antigen transport to distal immune-inductive sites, thereby further opening gateways to both mucosal and systemic immunity. In this context, slow-releasing gels like HPMC have proven counterproductive, ultimately compromising immune responses due to antigen masking. Conversely, enhancing antigen diffusion and mucosal absorption via permeation enhancers such as chitosan, appears key to preserving immunogenicity—thus enabling a more potent platform for sublingual vaccines. These findings contribute to the evolving framework for sublingual vaccine development and help guide the rational selection of excipients in future sublingual delivery systems.

## Figures and Tables

**Table 1:** Composition of sublingual vaccine formulations and summary of analytical tests performed on each formulation.

Formulation	OVA (µg)	CTB (µg)	MGC (µg)	HPMC (*w*/*v* %)	Immunogenicity	Sublingual Residence	Viscosity & Mucoadhesion
A	50	–	–	–	+	+	–
B	50	10	–	–	+	–	–
C	50	–	25	3	+	+	+
D	10	2	–	–	+	+	–
E	10	2	25	–	+	+	+
F	10	–	25	3	+	–	–
G	10	2	25	3	+	+	+

## Data Availability

The original contributions presented in this study are included in the article. Further inquiries can be directed to the corresponding authors.

## Abbreviations

The following abbreviations are used in this manuscript:

APCs	Antigen-presenting cells
AUC	Area under the curve
BSA	Bovine serum albumin
CTB	Cholera toxin B-subunit
EDTA	Ethylenediaminetetraacetic acid
ELISA	Enzyme-linked immunosorbent assay
FBS	Foetal bovine serum
GALT	Gut-associated lymphoid tissue
GMT	Geometric mean titre
GIT	Gastrointestinal tract
HPMC	Hydroxypropyl methylcellulose
HRP	Horseradish peroxidase
Ig	Immunoglobulin
IVIS	In vivo imaging system
MALT	Mucosa-associated lymphoid tissue
MGC	Methyl glycol chitosan
OVA	Ovalbumin
OD	Optical density
PBS	Phosphate-buffered saline
PBS-T	Phosphate-buffered saline with Tween 20
PIB	Protease inhibitor buffer
PG	Propylene glycol
ROI	Region of interest
TMB	3,3′,5,5′-Tetramethylbenzidine
VALT	Vulvovaginal-associated lymphoid tissues
